# Making Performance-Based Funding Work for Health

**DOI:** 10.1371/journal.pmed.0040219

**Published:** 2007-08-21

**Authors:** Daniel Low-Beer, Houtan Afkhami, Ryuichi Komatsu, Prerna Banati, Musoke Sempala, Itamar Katz, John Cutler, Paul Schumacher, Ronald Tran-Ba-Huy, Bernhard Schwartländer

## Abstract

Performance-based funding provides powerful incentives to scale up the fight against HIV, TB, and malaria, argues a team of authors from the Global Fund.

There have been significant increases in development finance since 2000, particularly for health [1,2]. As country programs put this money to work, important lessons and challenges on how to make health finance more effective emerge.

Performance-based funding provides clear incentives to achieve results and has been used by organizations such as the Global Fund to Fight AIDS, Tuberculosis and Malaria (the Global Fund) [3,4]. However, there are concerns that performance-based funding may penalize poorer countries and may not be flexible enough to contribute to health systems generally. Such concerns provoke two important questions: How are programs performing in countries at different levels of development, health systems strength, and disease burden? And what are the wider challenges of implementing performance-based funding to use health finances effectively, strengthen health systems, and achieve the Millennium Development Goals (MDGs)?

## Performance-Based Funding in Action: The Global Fund

To give an example, the Global Fund has disbursed US$3.24 billion to programs to fight HIV, tuberculosis (TB), and malaria in 130 countries and uses performance-based funding to evaluate program performance and manage grants. Funding is not guaranteed but is released based on demonstrated results against agreed country-owned targets and indicators that are set out in the initial grant agreement.

Supporting Information File S1 provides an extensive analysis of the results of 370 active grants to fight AIDS, TB, and malaria in 130 countries by programmatic service delivered, region, type of recipient, and country characteristics. Supporting Information File S2 gives a table of key data on grants reviewed with their ratings, principal recipient characteristics, disease component, and financial information. The analysis shown in File S1 assesses whether performance-based funding penalized poorer countries or those with weaker health systems. The results show the rapid scale-up of services to people in need, doubling each year, with financed programs providing 1.1 million people with HIV treatment, 2.8 million people with TB treatment, and 30 million people with insecticide-treated bednets (ITNs) to protect families from malaria.

Most importantly,75% of country programs reached their targets and were able to make the money work to deliver AIDS, TB, or malaria services. Of the remaining country programs, 21% had inadequate results but displayed the potential to accelerate implementation and achieve targets in the future, and 4% showed unacceptable results. Sub-Saharan Africa did not show substantially worse performance than other regions. The poorest third of countries performed no worse, indicating that performance-based funding works if targets are set and owned by the countries and set according to the country situation.

The analysis concludes that performance-based funding provides powerful incentives to scale up the fight against these three diseases by linking finance to the delivery of services. It is essential that targets are set and owned by countries rather than international funders, so that poorer countries, fragile states, and countries with weaker health systems are not penalized. But there are many challenges to successful performance-based funding, including how we ensure flexible “diagonal” funding so that “vertical” disease-specific initiatives on AIDS, TB, and malaria can also support general health systems.

## What Is Performance-Based Funding?

Driven by a commitment to ensure that health finance is effective, incentives are increasingly being used to focus on outcomes, identify problems, reward solutions, and manage results [1–4]. Performance-based funding was developed in the 1970s in the education sector, and is used by a number of recent development initiatives, including the GAVI Alliance (formerly known as the Global Alliance for Vaccines and Immunisation), the Millennium Challenge Account (see http://www.whitehouse.gov/infocus/developingnations/millennium.html), and the European Commission, as well as more general health initiatives [3–6].

The Global Fund uses performance-based funding to disburse all funds, which are released incrementally based on demonstrated results against targets, and fund recipients must provide an explanation of deviations, what has worked, and what can be improved. Explicit performance ratings are made for each grant and used as a basis for decisions, including: (1) stopping grants (which has occurred in Nigeria, South Africa, Senegal, and Pakistan); (2) reducing funding (the Kenya malaria program had US$5.8 million reduced commensurate with delays of one year); or (3) accelerating funding (the Ethiopia malaria grant had its year 4 budget accelerated into year 3 to double the number of ITNs distributed to families [Box 1]). Funds are geared to the speed and efficiency of implementation, not to a fixed calendar. If grant implementation is slower than planned, funds not used can be reallocated to other grants, and technical assistance needs can be addressed. If implementation is faster, funds can be accelerated.

Importantly, performance-based funding is based on radical country ownership of targets and implementation, with limited interference by the Global Fund as the donor. Implementation plans and targets are proposed by countries (with no formal involvement of the Global Fund), agreed upon by representatives of government, civil society, and people affected by the diseases in country (as part of a country coordinating mechanism), and reviewed by an international technical review panel. Performance is measured against what is realistic to achieve in country in a specific timescale. Russia is not, for example, pitted against Zambia, nor India against Swaziland. Country ownership and performance-based funding aim to strengthen basic country management. As the manager of the Guatemala programs commented, “If sub-recipients see their results falling into the red, they telephone us and we talk it through and find solutions. Equally important we feed back…our performance and we have a mature debate, not just on politics but implementation and progress, real grant issues” [5].

## Can Countries Make the Money Work?

There is considerable inherent variation and risk in the performance of financed programs for HIV, TB, and malaria. We often expect finance to “buy” results (as with the MDGs, which are costed at US$189 billion until 2015 [7]), and yet risks and returns are often not revealed until programs are implemented. In the Global Fund analysis (File S1), programs showing excellent performance achieved 120% of their initial targets for the main interventions, compared to 60% for programs with inadequate performance. Basing finance and technical assistance on differences in performance has the potential to accelerate implementation and double the results from health finance.

**Figure 1:**
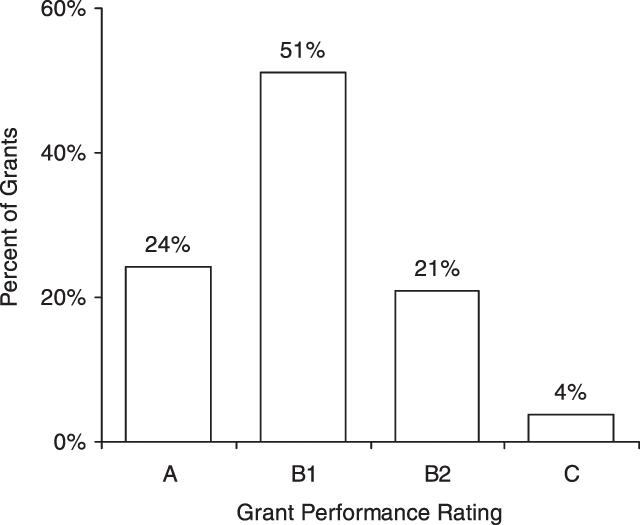
Variability in Performance of 215 Grants Evaluated by December 1, 2006 A: met or exceeded performance. B1: adequate performance. B2: inadequate performance but demonstrated potential. C: unacceptable performance.

**Figure 2:**
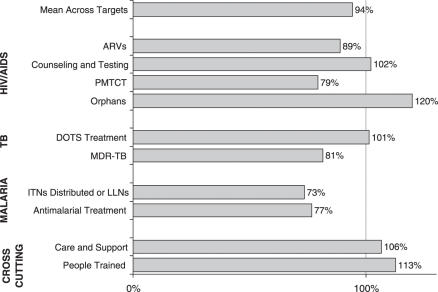
Variability in rogrammatic Performance in Major Service Areas for HIV/AIDS, TB, Malaria, and Cross-Cutting Care, Support, and Training ARV, antiretroviral; DOTS, directly observed treatment, short-course; LLN, long-lasting net; MDR-TB, multidrug-resistant tuberculosis; PMTCT, prevention of mother-to-child transmission.

Figure 1 shows that 75% of programs demonstrated satisfactory to excellent performance, 21% inadequate performance, and 4% unacceptable performance. The most difficult investment decisions were in the 21% of programs performing inadequately, due to the difficult trade-offs between poor performance against current targets and demonstrated potential to accelerate implementation. Taken together, the 215 programs evaluated for their first 18-month phase of implementation achieved 94% of their programmatic targets (Figure 2). Some of the programs fell below their individual targets, but well-performing programs allowed the Global Fund to achieve its results overall. Such well-performing programs allow the Global Fund to absorb a certain level of risk (for example, in poor performance of early investments in Nigeria, Uganda, and Kenya) and still achieve positive results overall. There were areas of weakness—for example, delays in the early stages of malaria interventions occurred because a few large grants in Ethiopia, Kenya, and Tanzania were not used quickly enough to distribute ITNs. But the example in Box 1 shows that a grant that is slow to get started can catch up rapidly once initial problems are solved and investments in capacity are made. The analysis also found that prevention of mother-to-child HIV transmission was underperforming more generally across many grants, and gender issues urgently need to be addressed to ensure that women are reached with what is essentially an affordable “vaccine” to prevent the infection of children. Despite many challenges, the majority of country programs can make the money work.

## Learning from Performance: Intelligent Implementation

Performance-based funding brings many of the challenges of implementation to the surface, allowing countries and partners to respond to them in a transparent manner, and promoting necessary “learning by doing.” There are several general lessons from program performance across the three diseases.

The analysis in File S1 showed that civil society was a particularly strong implementer (83% of civil society–implemented programs performed strongly), alongside its acknowledged role in governance and oversight. TB programs also performed particularly well (84% performed strongly), probably as a result of the important support of the Stop TB Partnership in coordinating partner roles clearly and providing a full package of managerial as well as technical assistance. Such partner support and formal coordination has clear lessons for HIV and malaria programs. Technical support was most needed when countries were scaling up services and showing results, as for example when the Malawi and Ethiopia programs extended successful services to rural areas. Such support is not just needed when problems arise. The greatest potential returns for technical support came from a general package to help adequately performing programs (as well as problem programs), which by our estimations can increase results in sub-Saharan Africa by 90% if effective. As mentioned, the poorest third of countries did not have worse performance ratings than wealthier countries. Only 9% of funds have been reallocated from these poorest countries due to inadequate performance, a third less than the funds removed from all grants. Performance-based funding can be an investment in performance and in reducing poverty.

## Conclusion: “Diagonal Financing” Can Support Health Systems

Despite many challenges, performance-based funding provides powerful incentives to scale up the fight against HIV, TB, and malaria. It also has more general implications for the delivery of development and health finance. At its best, performance-based funding combines the inventiveness of country solutions with the sharp focus and incentives of performance, ensuring people receive services with urgency. Most country programs have been able to make the money work, in poor countries and fragile states, across the three diseases and with civil society and government implementers. However, there is considerable inherent risk in the results we can expect from health programs and finance. The variability in returns in health programs (well-performing programs return twice the results) is not always recognized and needs to be actively managed with financial incentives and technical support.

A major question is whether funding for specific diseases like AIDS, TB, and malaria (“vertical financing”) has positive or negative effects on more general or “horizontal” health systems. The Global Fund provides “diagonal financing,” with a sharp focus on achieving disease goals while allowing finance to more broadly strengthen the supporting health sector. As health programs keep scaling up, the major gap in health systems strengthening is increasingly obvious. Such strengthening requires dedicated finances and increasing flexibility in disease-specific financing to fill key gaps in capacity. Due to the flexibility of Global Fund financing in supporting services and systems, countries with weaker health systems or human resources for health did not perform significantly worse (25% and 26% poor-performing compared to 25% for all grants). Global Fund finance can be delivered “vertically” to AIDS, TB, and malaria control programs or “horizontally” through pooled or sector-wide approach funding (for example in Ghana or Mozambique). Many effective programs have used Global Funds “diagonally” to effectively finance AIDS, TB, and malaria services, while building the necessary supporting systems, including human resources.

**Box 1: A Country Example of Performance-Based Funding: The Ethiopia Malaria Grant**

In June 2005, the Ministry of Health of Ethiopia had not yet delivered a single grant-supported ITN despite a target of two million ITNs to be delivered by this date. This lack of delivery was largely due to procurement bottlenecks and global supply-side problems, for both long-lasting ITNs and artemisinin-based combination therapy for malaria. The Global Fund placed very strict conditions on continued funding: funding would be discontinued if the ITNs were not delivered before the next malaria season.

The urgency of the conditions provided clear incentives for the country to come up with innovative solutions. Ethiopia requested technical support from UNICEF, increased their procurement office capacity, and invested to remove delays in the national supply chain. As a result, two million long-lasting ITNs were distributed within four months before the onset of the malaria season. The Ministry of Heath also was able to train 4,416 health workers on ITN use, train 5,222 health workers on malaria diagnosis and treatment, and deliver 2 million doses of artemisinin-based combination therapy.

Given this accelerated performance, the Ministry of Heath now intends to deliver an additional 7 million ITNs in line with achievement of the Abuja targets on malaria control (see http://www.rbm.who.int/docs/abuja_declaration_final.htm) and the MDGs. Given Ethiopia's current level of performance, the budget for year 4 has been accelerated into year 3. This is a good example of a malaria grant that can be slow to get started but catch up rapidly once initial problems are solved. The Ethiopian Minister of Health commented on the incentives of performance-based funding: “What made the difference is you gave us a clear warning, that we were in the red zone…we could lose our money if we didn't deliver results. We looked at it, we could focus and we both saw the problem…and that was the adjustment we made to get the results. Performance-based funding helped us think through implementation” [5].

On the ground, flexible finance is critical. When AIDS and malaria programs extended their services to rural areas of Ethiopia, they found that 30,000 community health workers needed training and community health posts needed refurbishing, which the Global Fund was able to support. In Haiti, a 14-month study showed that the Global Fund HIV program resulted in increased vaccinations, prenatal care, family planning, and flow of essential medicines, improving staff morale and health center capacity [8]. However, many programs do not use Global Fund finance as flexibly and effectively, and we need to “mind the gap” financially in health systems. Reaching the MDGs will require US$67 billion for human resources for health [7]. Other donors are urgently required, particularly for financing long-term, systematic health infrastructure [9]. If the MDGs are to be achieved, significant increases in finance and innovative mechanisms will be required to ensure that funds are delivered effectively. Performance-based funding may be one important mechanism to provide focus, incentives, and risk management in AIDS, TB, malaria, and health systems financing. 3

## Supporting Information

Text S1 Making performance based funding work: Progress and challenges of the Global Fund. (1.1 MB PDF).

Text S2 Table of key data on grants reviewed with their ratings, principal recipient characteristics, disease component, and financial information. (173 KB XLS).

Alternative Language Text S3 French translation of the article by R. Tran-Ba-Huy and E. Louis. (65 KB DOC).

## Abbreviations

ITN: insecticide-treated bednet
MDG: Millennium Development Goal
TB: tuberculosis

## References

[pmed-0040219-b001] World Health Organization (2005). Health and the Millennium Development Goals. http://www.who.int/mdg/publications/mdg_report/en/.

[pmed-0040219-b002] Organisation for Economic Co-Operation and Development (2006). Aid to health update 2006.

[pmed-0040219-b003] Lu CL, Michaud CM, Gakidou E, Khan K, Murray CJL (2006). Effect of the Global Alliance for Vaccines and Immunisation on diphtheria, tetanus, and pertussis vaccine coverage: An independent assessment. Lancet.

[pmed-0040219-b004] Porter ME, Teisberg EO (2006). Redefining health care: Creating value-based competition on results.

[pmed-0040219-b005] Global Fund to Fight AIDS, Tuberculosis and Malaria (2007). http://www1.theglobalfund.org/en/files/about/replenishment/oslo/Progress%20Report.pdf.

[pmed-0040219-b006] Global Fund to Fight AIDS, Tuberculosis and Malaria (2002). The framework document of the Global Fund to Fight AIDS, Tuberculosis and Malaria: Title, purpose, principles and scope of the fund. http://www.theglobalfund.org/en/files/publicdoc/Framework_uk.pdf.

[pmed-0040219-b007] UN Millennium Project (2005). Investing in development: A practical plan to achieve the Millennium Development Goals. Overview.

[pmed-0040219-b008] Walton DA, Farmer PE, Lambert W, Leandre F, Koenig SP (2004). Integrated HIV prevention and care strengthens primary health care: Lessons from rural Haiti. J Public Health Policy.

[pmed-0040219-b009] Shakow A (2006). Global Fund–World Bank HIV/AIDS programs: Comparative advantage study. http://siteresources.worldbank.org/INTHIVAIDS/Resources/375798-1103037153392/GFWBReportFinalVersion.pdf.

