# Supplementary material for: Making Performance-Based Funding Work for Health
Source: PLoS Med. 2007 Aug 21;4(8):e219. doi: 10.1371/journal.pmed.0040219 (PMC1949844; doi:10.1371/journal.pmed.0040219)
Supplement: Supplementary file 1 [file pmed.0040219.sd001.doc]

**Making performance based funding work:**

**Progress and challenges of the Global Fund**

Daniel Low-Beer, Houtan Afkhami, Ryuichi Komatsu, Prerna Banati, Musoke Sempala Itamar Katz, John Cutler, Paul Schumacher, Ronald Tran-Ba-Huy, Bernhard Schwartlander

Corresponding Author: Daniel Low-Beer, daniel.low-beer@theglobalfund.org

Affiliations: Global Fund to Fight AIDS, TB and Malaria, Chemin de Blandonnet 8, 1214 Vernier, Geneva, Switzerland. Low-Beer also Cambridge University Health, Judge Business School, Cambridge University, UK.

Additional author contacts: Houtan Afkhami ([Houtan.afkhami@theglobalfund.org](mailto:Houtan.afkhami@theglobalfund.org)); Ryuichi Komatsu ([Ryuichi.komatsu@theglobalfund.org](mailto:Ryuichi.komatsu@theglobalfund.org)); Prerna Banati ([prerna.banati@theglobalfund.org](mailto:prerna.banati@theglobalfund.org)); Itamar Katz ([Itamar.katz@theglobalfund.org](mailto:Itamar.katz@theglobalfund.org)); John Cutler ([john.cutler@theglobalfund.org](mailto:john.cutler@theglobalfund.org)); Musoke Sempala (Musoke.sempala@theglobalfund.org); Paul Schumacher ([Paul.schumacher@theglobalfund.org](mailto:Paul.schumacher@theglobalfund.org)); Ronald Tran-Ba-Huy (Ronald [tran-ba-huy@theglobalfund.org](mailto:tran-ba-huy@theglobalfund.org)); Bernhard Schwartlander ([Bernhard.schwartlander@theglobalfund.org](mailto:Bernhard.schwartlander@theglobalfund.org))

**Abstract**

**Background**: The Global Fund has disbursed US$ 3.24 billion to programs to fight HIV, TB and Malaria in 130 countries. It implements performance based funding to explicitly evaluate the performance of programs, and manage grants. This paper assesses the performance of grants by programmatic area, region, type of recipient, poverty and health systems. Financial analysis of decisions to continue funding against grant performance is presented, as well as the first substantial analysis of the programmatic results of the portfolio. The challenges of implementing performance based funding are discussed.

**Methods and Findings**: Performance of results reported to the Global Fund against targets for all 370 active Global Fund grants by end 2006 were aggregated by key programmatic area, and compared over time. Variations in performance of the 215 grants which have been evaluated for their first phase of implementation were analysed by programmatic area, region, type of recipient, and country characteristics. Financial commitments to grants following performance evaluation were also analysed overall and to assess if performance based funding penalized poorer countries or those with weaker health systems. Despite variations in performance, Global Fund financed programs provided 1,100,000 people with ARV treatment, 2.8 million people with TB treatment under DOTS, and 30 million insecticide treated bed nets to protect families from malaria. Most importantly, 75% of the 215 country programs which have had their first phase of funding evaluated show satisfactory to excellent performance. Overall 94% of programmatic targets were achieved, with considerable variability from 73-120%. Civil society is a strong implementer, as are TB grants, and grants in Sub-Saharan Africa have no worse performance. Performance based funding does not appear to penalize countries with lower income or poorer health systems.

**Conclusions**: Despite many challenges, performance based funding provides powerful incentives to scale up the fight against the three diseases, linking finance to the delivery of services. The majority of grants are performing, in poor countries, across the three diseases, and with a variety of grant recipients. The basis of country owned and proposed targets for performance evaluation is essential so that poorer countries, fragile states and those with weaker health systems are not penalized. However there is considerable variety in performance, and improved efforts to support grants with technical assistance is required, including an additional focus to support adequately performing grants (rather than just the poorest performers).

**Introduction**

The Global Fund to fight AIDS, TB and Malaria was set up with the ambitious goals to impact the three diseases and contribute to the collective Millennium Development Goals (MDGs) [1,2]. The Global Fund also developed from the movement to harmonise and improve the accountability of donor efforts and ensure country ownership in the delivery of aid [3]. This is reflected in the seven principles which guide its policies and operations (Box 1) [4]:

**Guiding Principles of the Global Fund**

**The Global Fund:**

- **Operates as a financial instrument, not an implementing entity.**
- **Makes available and leverages additional financial resources.**
- **Supports programs that evolve from national plans and priorities.**
- **Operates in a balanced manner with respect to different geographical regions, diseases and healthcare interventions.**
- **Pursues an integrated and balanced approach to treatment, care and support.**
- **Evaluates proposal through an independent review process.**
- **Operates transparently and accountably and employs a simplified, rapid and innovative grant-making process.**

***Box 1: Guiding Principles of the Global Fund***

As well as fighting AIDS, TB, the Global Fund’s model of disbursing funds is of wider relevance to debates on aid effectiveness and health [5-7]. First, the Global Fund relies on implementation plans proposed by countries (with no involvement of the Global Fund), agreed by representatives of government, civil society and people affected by the diseases in country (as part of a country coordinating mechanism). Secondly performance based funding is used to disburse funding – funding is not guaranteed but released based on demonstrated results against agreed targets. Performance based funding is used by a number of recent health and development initiatives, including GAVI (the Global Alliance for Vaccines and Immunisation), the Millennium Challenge Account, the European Commission, as well as more generally in health [8,9].

However the Global Fund’s approach to performance based funding combines country ownership with a performance based funding system for receiving, managing and monitoring investments based on the principles of accountability and transparency. Explicit performance decisions and ratings are made on each grant, including stopping grants or reducing funding, for example in Nigeria, South Africa, Senegal, and Pakistan. Performance based funding provides considerable information to assess the progress of health and development investments by programmatic area, region, type of recipient and country characteristics (including poverty and health systems) of wide relevance to health development. The Global Fund implements performance-based funding using country owned targets and indicators as set out in the initial proposal and grant agreement. Funds are disbursed incrementally based on program performance in meeting these targets. The typical proposal is approved for a period of five years. This period is divided into two phases: initially, funds are committed for the first two years (‘Phase 1”), after which its performance is evaluated through an extensive review process and appropriate funds committed typically for the remaining three years (“Phase 2”).

Funding is related to the speed and efficiency of implementation rather than to a fixed calendar. If a grant implements quickly funding can be accelerated, if it implements more slowly funds not used can be reallocated to other grants, and technical assistance needs are flagged. Performance based funding provides powerful incentives to identify problems, reward solutions and boost implementation. Overall, the aim is to ensure the money works transparently for those in need of services.

The purpose of this paper is to present the results and assess the performance of Global Fund grants to mid-2006. It aims to analyse performance by disease, region, type of recipient and country characteristics, and present some of the challenges to implementing performance based funding.

**Methods**

Performance of results against targets for all 370 active Global Fund grants by end 2006 were aggregated by key programmatic area, and compared over time. Results are reported to the Global Fund before each disbursement of funds, and verified in country by the Local Fund Agent of the Global Fund. The results for grants were aggregated to 1^st^ Dec, 2006, based on data which is publicly available on the Global Fund website for each grant ([www.theglobalfund.org](http://www.theglobalfund.org/)). In addition, the speed of financial disbursements to the grants was assessed by comparing the amount disbursed to what would be expected by the time elapsed in these grants. This assumed a linear relationship between financial disbursement and time elapsed in grants, which is appropriate over all grants though may vary significantly for an individual grant.

Variations in performance of the 215 grants which had been evaluated for their first phase of implementation (phase 2 evaluation) by 1^st^ Dec, 2006 were analysed. Results were aggregated by major programmatic area and compared as a percentage of the sum of their targets, and the mean performance across these programmatic areas calculated. This method was used to ensure that overall performance reflected the contribution of very different sized grants. Changes to overall results were assessed by comparing results against targets for A, B1, B2 and C rated grants, to assess the marginal increase in overall results if grants performed at a higher level. The indicators used are agreed by major partners, and published in the multi-partner monitoring and evaluation toolkit [10]. In addition variations in individual grant performance were analysed by disease, region, type of recipient, and country characteristics including poverty and health systems. Civil society includes non-government, faith based, and private sector organizations, ranging from the Churches Health Association of Zambia, to Sogebank, the non-profit grant giving arm of the largest private bank in Haiti. Grants are given an explicit performance rating based on their results against targets (A-rated achieved 80% of targets, B1-rated grants 50-79% of targets, B2-rated grants 30-49% of targets, C-rated grants less than 30% of targets). Contextual factors including the quality of data are included in the end evaluation rating, which is provided explicitly with a grant scorecard of all data used for the decision on the Global Fund website (http://www.theglobalfund.org/en/funds_raised/gsc/). The overall evaluation rating is reviewed by the Local Fund Agent in country, by two units independently in the Global Fund Secretariat, with a final performance decision made by a panel in the Secretariat and a funding decision by the Board of the Global Fund. The rating is based on results against targets with the cut off ranges provided above, but there are important trade-offs with contextual data. A-rated grants “meet or exceed expectations”, B1-rated grants show “adequate performance”, however contextual information is particularly important for B2-rated grants which show “inadequate performance but demonstrated potential”, and C-rated grants which have “unacceptable performance”.

For example the Ethiopia Malaria grant described later, achieved 0% of its crucial ITN distribution targets, but 100% of its ITN training and 95% of malaria diagnosis training targets. It had taken demonstrated steps to identify and solve procurement problems including using UNICEF and WHO. It was given a B2 rating (inadequate performance but demonstrated potential), with the condition that it catches up ITN results within 6 months (full data and explanation of this decision is provided on the Ethiopia malaria Round 2 grant scorecard on the web). The Senegal malaria grant (Round 1) was given a C rating, as it had not achieved antimalarial treatment or malaria in pregnancy targets. In addition it had disbursed only 36% of grant funds, not yet entered into contracts with sub-recipients after two years, and had acute governance problems between government, civil society and the private sector in its coordination body. The C rating involved a judgment on potential alongside results. The Senegal malaria grant was not recommended for continued funding.

There are critiques of performance based funding, including that it provides a narrow focus on results and on the three diseases, that the quality of country data is uncertain, and that it may penalize poorer countries or those with weaker health systems [1,2,5,7]. Poorer countries may be penalized if they are not allocated sufficient funds initially at proposal review, in the setting of their targets, or if funds are removed during grant implementation due to performance based funding. This paper assesses the per capita grant commitments in poorer countries and countries with weaker health systems (excluding the outliers of India and China where grants are in particular districts in large, populated countries), but focuses on whether these countries perform worse and lose funds during implementation. To assess poverty and health systems, grants were divided into three equal categories (low, middle, high) in terms of income (GNI per capita), human resources for health [11] and health systems performance [12]. These were based on WHO definitions, with health systems capacity measuring health outcomes, health system responsiveness and equity, and human resources for health ranking based on all health personnel (including doctors, nurses, and pharmacists) per capita. Grant performance was analysed by these criteria, as were funding commitments made for the second phase of implementation. The aim was to provide evidence as to whether performance based funding penalized poorer countries or those with weaker health systems during implementation. The paper provides the first substantial descriptive analysis of programmatic results and performance from Global Fund grants, providing analysis of the variations and challenges.

There are important limitations to the data, including well known data quality issues in results from country monitoring and evaluation systems, variations between countries, and limitations to the classification criteria for income and health systems resources. The paper also provides transparent descriptive analysis, and would benefit from a longer time series of performance data to assess if differences continue to be significant.

**Results**

In the early stages of the Global Fund, progress was measured by the number of grants signed and funds disbursed [13]. The average age of grants is still young, only 23 months as of 1^st^ Dec, 2006. In just over three years, the Global Fund has disbursed US$ 3.24 billion to 130 countries. Despite delays in individual grants, overall funds across all grants have been disbursed relatively quickly, 95% of grant money has been disbursed compared to the time passed in grants (on average grants are 71% through their grant time, and have spent 67% of their money). In addition, 81% of money disbursed by the Global Fund has been expended by the end user. There are exceptions to this general trend, for example grant spending in Uganda has been delayed due to complicated implementation arrangements between the Ministry of Finance, Ministry of Health and a separate country implementation unit initially established (and later disbanded) to disburse the grant. Grant disbursements in Ukraine, Myanmar and Uganda were also suspended, but for the majority of grants funds are disbursed and expended relatively quickly.

Increasingly there is a wide range of grant results and performance data, in addition to financial information. To 1^st^ June, 2007 results consolidated from the entire portfolio of grants supported by the Global Fund show:

- 1,100,000 people currently on antiretroviral treatment to treat AIDS
- 2.8 million people with TB treatment under DOTS
- 30 million insecticide treated bednets (ITNs) to protect families from malaria
- 17.8 million reached with HIV counseling and testing
- 53 million people reached with community outreach services
- 28 million people treated for malaria
- Trained 4.7 million additional service deliverers to fight HIV, TB or malaria

These are the results of the work of countries, supported by technical assistance from partners, for which the Global Fund only provides financing. The results are not fully attributed to Global Fund finances, for example the Malawi program has people on ARV treatment with funding from the Global Fund, additional donor funding including for health personnel from DFID and the SWAp, a Global Fund health systems strengthening grant, and critical technical support from CDC, World Bank, WHO, and UNAIDS. The work is completed by the government, NGO and local partners, with AIDS coordinators appointed in 15 key ministries and 28 districts, and support from the government recurring budget. The results are from programs, and their performance is assessed as a country program, and not attributable directly to Global Fund finance.

In addition, the time trend of results (by their date of reporting to the Global Fund) shows rapid scale up of services to mid-2007, increasing more than two times since the same time the previous year. Increases in the last year were 100-165%. The regional distribution of results is also shown.

***Figure 1 (a) Global Fund mid-2007 results from all grants for ARVs, ITNs distributed and TB treatment under DOTS (b) Map of distribution of results by region***

There are many challenges to this stage of accelerating results, and it is therefore important to assess the variability in performance of global fund grants. Performance based funding brings many of these country challenges to the surface, so countries and partners can respond. Current results, despite showing significant levels of services delivery and growth, fall far short of results needed to match the threat of the three diseases. The fuller analysis of these results and against international targets is provided elsewhere (see Global Fund progress report [3]).

There has been considerable variation in the performance of Global Fund financed programs. 215 grants have been evaluated for their first phase of implementation and given explicit performance ratings based on results against their targets (Phase 2 evaluation) by 1^st^ Dec, 2006. They provide a very important window into the performance and challenges of Global Fund investments. Seventy five percent of grants showed satisfactory to excellent performance (A or B1 rated), 21% showed inadequate performance but documented potential (rated B2), and 4% showed unacceptable performance (rated C). The most difficult investment decisions were in the 21% of grants rated B2, due to the difficult trade-offs between performance against current targets and demonstrated potential. Honduras HIV, TB and Malaria grants were given a B2 rating due to problems with performance, in addition to serious coordination problems between government and civil society most affecting the HIV grant (which was not recommended for continued funding). Strong incentives and technical support are required to increase the percentage of A-rated grants (21% of 215 grants), as this is where rapid acceleration of service delivery can be released. If B1 grants were supported to perform as A-rated, overall global fund results would increase by 90%.

***Figure 2: Variability in performance of 215 grants evaluated by 1^st^ Dec, 2006***

Taken together these 215 grants achieved 94% of the sum of their programmatic targets, with a range of 73 to 120% for the main services provided. Importantly, this shows that despite individual grant variation, collective targets are reached across the portfolio (the overachievement of high-performing grants, for example the China TB grant reached 115% of its treatment targets, compensating for several smaller, underperforming grants. Similarly the Ethiopia malaria grant was accelerated in year 3 to achieve targets early). This is an essential element of performance based funding, ensuring overall portfolio performance is strengthened by relating funding to performance. Performance was lower for malaria indicators due to procurement delays at the early stages of some grants. The Tanzania Malaria grant was delayed by over a year due to procurement complications with its voucher scheme for distributing ITNs (which used private sector networks to deliver the nets), as was the Kenya Malaria grant which required the establishment of a third party procurement agency. However, when the problems in malaria grants were overcome, results tended to catch up rapidly (see Box 2 for an illustration of performance issues in country grants). The rapid increase in ITNs delivered in the last year (165%) to 30 million, shows the strong returns from initial investments in country capacity for malaria by the Global Fund.

***Figure 3: Variability in programmatic performance for major service areas***

**Box 2 A country example of performance-based funding: Ethiopia malaria grant.**

In June, 2005, the Ministry of Health of Ethiopia, the principal recipient of the Global Fund grant, had not yet delivered a single grant-supported ITN despite a target of two million ITNs to be delivered by this date. This was largely due to procurement bottlenecks and global supply-side problems, for both long lasting ITNs and ACT treatment. The Global Fund placed very strict conditions on continued funding, that funding would be discontinued if the ITNs were not delivered before the next malaria season.

The urgency of the conditions provided clear incentives for the country to come up with innovative solutions to solve the problems that were slowing program implementation. They also requested technical support from UNICEF, increased their procurement office capacity, and invested to remove delays in the national supply chain. The results demanded by performance-based funding provided major incentives to focus efforts on overcoming the implementation bottlenecks. As a result, two million long-lasting ITNs were distributed within 4 months before the onset of the malaria season. The Ministry of Heath also was able to train 4,416 health workers on ITN use, train 5,222 health workers on malaria diagnosis and treatment, and deliver 2 million ACT doses.

Given accelerated performance, the grant now intents to deliver an additional seven million ITNs in 2006 in line with achievement of the Abuja target and millennium development goals of 60 percent coverage of the population at risk. Given its current level of performance, the budget for 2006 has been accelerated by the Global Fund to allow rapid scale-up of the response to achieve impact. This is a good example of a malaria grant which can be slow to get started, but can then catch up rapidly once initial problems are solved. The Ethiopian Minister of Health commented on the incentives of performance based funding: “*What made the difference is that you gave us a clear warning that we were in the red zone, that we could lose our money if we didn’t deliver results. We looked at it, we could focus and we both saw the problem, and that was the adjustment we made to get the results. Performance-based funding helped us think through implementation*” [3].

At all stages in the grant lifecycle, funding decisions are made on the basis of performance data, contextual explanation and an explicit evaluation. The financial investments in grants after Phase 2 evaluation is shown in the table below. This shows the original proposal amount for grants, the Board Confirmed Amount after performance review of the first phase of implementation, and the amount and percentage of funds released due to terminated grants and budget cuts in continued grants. Phase 2 funding has shown itself to be a significant investment in performance, while releasing finances to fund new grants.

***Figure 4: Financial status of Phase 2 decisions by 1^st^ Dec, 2006 showing original Proposal amounts, and confirmed amounts for the 215 grants evaluated for Phase 2, value of terminated grants and budget cuts and reallocated funds.***

Overall, US$ 2.2 billion has been committed to grants for Phase 2 based on performance, almost the equivalent of two new rounds of financing. A total of US$ 345 million in requested funding (13.5% of the total proposed) was reallocated to new grants, either due to budget efficiencies or grants discontinued due to poor performance. The Thailand HIV grant, for example, due to the successful national universal access health care plan, and declining prevalence in the targeted groups, was able to save US$40 million, which was returned to the Global Fund. Other grants for example the Kenya malaria grants, had US$5.8 million reduced from their budget at Phase 2 evaluation, commensurate with the implementation delays of one year. Reallocated funds were reinvested immediately, and financed the equivalent of one-third of new annual grant proposals in recent rounds of funding. Overall grants with A-rated performance received almost all of their funding, B1-rated grants over 90%, B2 rated 66% and C-rated grants less than 15%.

Phase 2 evaluation is only one point in performance based funding. Funding decisions are made on the basis of performance throughout the grant lifecycle, disbursement by disbursement. These involve a self assessment of reasons for deviations from targets, delays, and important contextual factors which are taken into account in the final investment decision (as shown in grant scorecards). It is crucial that performance data based on country owned targets is used actively at all levels to support decisions. To compare to the findings from phase 2 evaluation, Figure 5 also shows the percentage of grant amounts disbursed by performance rating for disbursements. They show consistent trends with A-rated grants receiving more than expected disbursements (based on the time elapsed in these grants), B1-rated grants 96 percent, and B2/C-rated grants below expected at 85%. There are arguments that this gradient should be more pronounced for disbursements, given that at phase 2 decisions B2-rated grants received 66 percent of phase 2 amounts, and C-rated grants only 14 percent.

***Figure 5: Expected finance disbursed against grant performance rating of grants at Phase 2 Evaluation and for the latest disbursement (end of 2006)***

The overall analysis of phase 2 grants shows considerable variation in performance, which it is important to analyse by grant and country characteristics. Particular attention is required to ensure over time that performance based funding does not penalize poorer countries or those with weaker health systems. Variations in grant performance by disease, region and principal recipient type (government, civil society, UNDP) is shown below for the 215 grants which were evaluated for their first phase of implementation by 1^st^ Dec, 2006.

***Figure 6 Variations in grant performance by a) disease, b) region and c) principal recipient type for the 215 grants which have been evaluated by 1^st^ Dec, 2006.***

Variations in performance show that civil society is a strong implementer (83% are A or B1-rated), as are TB grants (83% A or B1-rated), and sub-Saharan Africa does not have substantially worse performance (69% A or B-rated) than other regions. However Sub-Saharan Africa does have somewhat fewer A-rated grants (22% compared to 24% overall). Civil society organizations are important implementers alongside governments. TB grants are strong performers. There may be important lessons provided by the comprehensive technical assistance package provided by the Stop TB Partnership, which is not available for HIV or malaria. The Global TB drug facility also plays a significant role in reducing procurement delays.

Importantly, analysis showed that performance based funding does not appear to penalize poorer countries or those with weaker health systems. Only 20% of the lowest third of grants in terms of income were rated B2 or C for performance, compared to 30% in the middle and 25% in the highest income categories. Only 9% of funding in the poorest countries could not be used and was reallocated at Phase 2, compared to 19% for middle and higher income countries. Countries with the lowest capacity of human resources for health were also not penalized by performance based funding (19% rated B2/C compared to 25% in other countries). However countries with the highest level of health systems performance do perform slightly better (17% rated B2/C compared to 33% and 25%). There may be areas of health systems that require additional attention in grants, and certainly systematic health systems funding including of long, term human resource plans are required from other donors (for example the World Bank, and in Malawi where DFID has provided additional support of over UK£100 million for health human resources). Per capita disbursements were higher for poorer countries, 1.64 US$ per capita compared to 0.83 US$ for other countries, and for countries with the lowest health systems capacity (2.18 US$ compared to 0.55 US$). Furthermore, performance based funding during implementation does not reduce the amount of funds committed to these countries, and for poorer countries the reverse may be true. The trend may not however be linear. The poorest countries with weakest health systems may not be disadvantaged, but countries with the strongest health systems do appear to have performance advantages.

**Figure 7 Grant performance by levels of wealth, health systems strength and human resources for health divided into equal categories (n= 69 grants in lowest wealth category, 68 for health systems and 70 for human resources for health). Percentage of grants rated B2/C by equal categories (low, middle, high) of wealth (GNI per capita), health systems capacity (based on WHO 2000 World Health Report, Statistical Annex 10, [11]) and human resources for health capacity (WHO 2006 World Health Report, Statistical Annex 4, [12]).**

**Conclusion: the challenges of performance based funding**

In the last four years the Global Fund has financed the scale up of services for HIV, TB and malaria through performance based funding, an important innovation in delivering development finance [3,14]. The approach is based on country owned implementation arrangements and targets. At its best, performance based funding combines the inventiveness of country solutions with the sharp focus on performance, ensuring people receive services with urgency. There are also critiques that performance based funding encourages a narrow focus on results and on the three diseases, of the quality of country data, and that it may penalize poorer countries and those with weaker health systems. The majority of grants are still young, but this paper has provided information on the performance of grants and programmatic results.

There are significant variations in performance, but taken together Global Fund financed programs provided 1,100,000 people with ARV treatment, 2.8 million people with TB treatment under DOTS, and 30 million insecticide treated bed nets to protect families from malaria. Most importantly, 75% of the 215 country programs which have had their first phase of funding evaluated, show satisfactory to excellent performance, suggesting the majority of countries can make the money work, despite well documented problems in some countries for example Uganda, Myanmar or Ukraine. Overall 94% of programmatic targets were achieved, with considerable variability from 73-120%. Civil society is a strong implementer, as are TB grants. Grants in Sub-Saharan Africa have no worse performance (B2 or C rated), but somewhat fewer excellently performing A-rated grants. Performance based funding does not appear to significantly penalize countries with the lowest income or poorest health systems.

There are important limitations to the data and analysis, including well known data quality issues in results from country monitoring and evaluation systems, variations between countries, and limitations to the classification criteria for income and health systems resources. There are also limitations for descriptive analysis to the sample size of 215 grants evaluated for their first phase of implementation, including a bias towards grants from earlier rounds of funding which may not reflect recent trends in portfolio performance. Nevertheless, the paper provides significant descriptive analysis of aggregated programmatic results from grants, and analysis of variations in grant performance. The analysis would benefit from continued verification against grant results over time, and additional analysis of some of the key themes identified in this paper using multivariate analysis. Some of the performance differences may require observation over a longer time period to be substantiated as significant, for example of the impact of health systems and differences by region.

In addition there are limitations to the quality of data in countries, and the targets used as the basis for performance evaluation require further analysis. The Global Fund does undertake assessments of country M&E systems at the start of a grant (including using 5-10% of grant funds to strengthen data quality), independent reviews of data by Local Fund Agents including site verifications each year, and is undertaking data quality audits on a sample of grants (which will be carried out by organization not involved in implementation to avoid conflicts of interest in country). Data quality issues have affected some grants, and for example in the Nigeria HIV grant (where ARV patient results could not be verified) have contributed to discontinuation of funding. In addition, the quality of results vary by programmatic indicator, quantitative indicators of treatment, and ITNs distributed are more clearly measured than many prevention and care indicators. Targets are proposed by countries and reviewed by an independent technical review panel (which rejects over 50% of proposals) as well as by the Secretariat. They are influenced by international targets (for example Round 4 country HIV treatment targets were strongly boosted by the momentum of WHO’s 3 by 5 program, and Round 6 grants by the new WHO Stop TB strategy). Targets are ultimately proposed and accepted in relation to what is realistic in countries. For poorer countries not to be penalized it is important that they set realistic targets. However, as has been the case in Tanzania, Ethiopia and Malawi where targets have been increased, they should also tackle the full scale of the epidemic in rural areas and in every district as part of a national program. Targets set in the proposal should not decrease in grant agreements (and in some countries for example Ethiopia, Tanzania have been revised upwards during implementation), but despite checks some slippage may occur. The assessment of targets and the various incentives involved in countries and internationally, requires further analysis.

The analysis does show the strength of civil society as an implementer, alongside its role in governance and oversight. It may be important to ensure there is a direct track of finance to civil society alongside government (dual track financing), which may improve the absorptive capacity of countries to use funds rapidly. In Zambia, the involvement of the Churches Health Association as Principal Recipient for Malaria, TB and HIV grants alongside government and other civil society organizations has greatly increased the institutional and human resource capacity (given important public sector capacity constraints), and the speed of disbursement which can be made directly to civil society. Problems of coordination still remain, for example delays in government procurement have slowed all grants in Zambia. Civil society can also play a primary role, for example in Haiti Sogebank (the largest private bank) has been central to delivering HIV, TB and Malaria grants, using its management and financial experience to coordinate multiple sub-grantees from government, NGOs and donors in a fragmented and fragile health system. Similarly in Ukraine after the suspension of the grants in 2004 related to the government, the International HIV/AIDS Alliance managed the grants successfully under a Stewardship agreement, achieving full phase 2 funding for the country. National plans may not always have been devised to include full civil society participation and coverage of key groups, for example sex workers, drug users, and full participation of civil society from national ownership to implementation is important.

In Sub-Saharan Africa, an additional strategy to focus technical assistance on B1-rated grants, in order to accelerate performance to become A-rated, may have significant returns, alongside the traditional focus on poorest performers. Many countries need technical assistance most when they are scaling up implementation and show results, not just when problems arise. In Malawi, Tanzania and Ethiopia it is when the grants were performing and pushing scale up of HIV treatment to the rural areas that technical assistance needs were most acute, and were provided by DFID in human resources, the World Bank for Monitoring and Evaluation, and by reprogramming the existing Global Fund grant after two years to release $US 40 million for health human resources (alongside DFID). In Ethiopia 30,000 primary health workers are being deployed with Global Fund finance, together with World Bank and GAVI support, with CDC and USAID providing technical support to health centers. Overall, our analysis suggests that if B1 grants could be supported to perform as A-rated, technical assistance would have its greatest returns with overall global fund results potentially increasing by 90% if such strategies were effective.

There have also been many challenges to implementing performance based funding. First, it was essential to base it on country owned targets, what is seen as realistic by those implementing programs, yet evaluated independently by a technical review panel. This ensured country ownership and innovation throughout the system, and avoided the top down approach to performance measurement which can stifle ownership and flexibility. Secondly the Global Fund developed increasingly open monitoring and evaluation systems. It brought together partners to agree on common indicators for the three diseases rather than developing specific Global Fund indicators. This has allowed performance based funding to provide incentives for harmonisation, using existing indicators and national systems. The open M&E approach supports the Three Ones Principles for the three diseases (in particular a common national M&E system to produce results: as described for AIDS [15,16]). Thirdly, it was important that performance data was used actively for decisions throughout the grant lifecycle, in planning, Phase 2 decisions, and each disbursement. There were many challenges in individual decisions (in particular the first 215 Phase 2 decisions) but overall funding has followed performance. The overall results show rapid scaling up of results, doubling annually. This is based on focused investments, geared to the pace of implementation of country programs. The majority of countries can make performance based funding work, independent of levels of poverty, health systems or fragile states [3,5].

Performance-based funding also brings many of the challenges of implementation to the surface. This should allow countries and partners to respond to them in a transparent manner based on documented evidence. There have been many initial procurement delays in malaria grants, often due to global supply bottlenecks for example in long lasting bed nets and ACT drugs. A major obstacle to ARV enrollment (and particularly PMTCT losing many women after their initial clinic visit) has been complex HIV testing and counseling procedures. This has been radically simplified in Malawi and Botswana grants in terms of procedures for testing, pre and post test counseling and promotion of more routine testing (with the possibility of opting out). The pricing for ARV drugs is also critical for sustainability, particularly with second line treatment and drug resistance [17]. Management and financial capacity is a major bottleneck for all three diseases, in addition to traditional technical health assistance. Other sources of technical assistance, for example from NGOs and the private sector, Sogebank in Haiti, the medical research council in South Africa which coordinates malaria grants in Swaziland and Mozambique, may be required. Grants need to allocate sufficient grant resources for management and financial capacity, and use technical assistance from public and private sources where needed. Performance-based funding has contributed to improving transparency and accountability [18-24]. In several grants, Nigeria with data problems, Kenya with weak financial systems, Ukraine with grant suspension, problems have been raised, so where feasible they can be solved. Data and explanations of performance are available, evaluated actively, and increasingly used by all partners to respond to country problems.

This paper substantiates previous analysis based on financial data [5,27,28] suggesting the large majority of programs, including in poorer countries, can convert large amounts of finance into programmatic performance. Only 20% of the lowest income countries had poor programmatic performance, half that of other countries. In addition only 9% of their funding could not be used and was reallocated. However some weaknesses are apparent, not shown from financial data [5]. Sub-Saharan Africa has fewer A-rated over performing programs. A new technical assistance strategy to support B1 rated grants in Sub-Saharan Africa to accelerate, will have the greatest returns. Human Resources capacity can be improved directly by grants, in Malawi US$40 million was reprogrammed two years into implementation due to concerns raise by performance evaluation at phase 2, to fund the human resources gap to deliver HIV services. This was further supported by a Global Fund Health Systems Strengthening grant funding the health systems strategy, including training and a medical school. An equally important human resources capacity strengthening element was the provision of ARV treatment to health care workers, a major source of attrition (together with outmigration) in the 1990s. However this strategy required much more extensive donor support, in Malawi from DFID. In addition it is essential they are better linked to longer term human resources plans, and despite problems this has been improving with successive Global Fund rounds of proposal [25]. Others areas of health systems capacity are not so easily financed, and systematic, long term development of fundamental health infrastructure is required from other donors, for example the World Bank [26].. It is important to monitor that Global Fund grants support service delivery and related capacity building (48% of funding is committed to commodities, 52% to capacity building including human resources, clinics and laboratory equipment) [3] to continue to absorb large scale financing. Performance based funding is a potential tool to manage absorptive capacity, linking financing to the speed at which these funds can be absorbed (increasing or decreasing the amount depending on the rate of absorption and results). At present, there are few signs of diminishing marginal returns overall, financially or programmatically, although systematic investment in health systems is urgently required. Initial investments in the three diseases that grip the African health sector may even release health resources in some situations which are limited and local at present, as described by an African Minister of Health describing the impact of AIDS treatment in some areas “*You go to the medical ward and now half of the beds are not occupied, before they were mushrooming. A manager came to me as Minister of Health and said “You are bad for business, our funeral business is going down. There was a time when every weekend we were burying four to eight people, now weeks go by without a funeral”*[3].

The results and challenges go far beyond what can be achieved by one organization, and are the results of the work of countries, international and local partners. The Global Fund is a network or partnership organization and needs to continue to mobilize a response at international, national and community level to deliver results. It needs to work closely with partners to continue to improve the wider investment conditions for health in countries, including longer term, costed, national health plans, better links to development plans, and improved financial, accountability and supply chain systems. Systematic investment in health systems by other donors, including health personnel, is critical to further accelerate programme scale-up [27,28]. Progress has been remarkable, but there is a long way to go to reach international targets and the millennium development goals in 2015. Performance based funding has proved an important mechanism to finance the fight against HIV, TB and malaria.

**References**

1. Brugha R, Donoghue M, Starling M, Ndubani P, Ssengooba F, et al. (2004) The Global Fund: managing great expectations. Lancet 364:95-100.

2. Brugha R, Starling M, Walt G (2002) GAVI, the first steps: lessons for the Global Fund. Lancet 359:435-8.

3. Global Fund to Fight AIDS, Tuberculosis and Malaria (2006) Partners in Impact: Results Report. Geneva: Global Fund to Fight AIDS, Tuberculosis, and Malaria.

4. Global Fund to Fight AIDS, Tuberculosis and Malaria (2002) The framework document of the Global Fund to Fight AIDS, Tuberculosis and Malaria: title, purpose, principles and scope of the fund. Geneva: Global Fund to Fight AIDS, Tuberculosis, and Malaria.

5. Lu C, Michaud CM, Khan K, Murray C (2006) Absorptive capacity and disbursements by the Global Fund to Fight AIDS, Tuberculosis and Malaria: analysis of grant implementation. Lancet 368:483-88.

6. Sachs J (2001) Macroeconomics and health: investing in health for economic development. Geneva: WHO.

7. Radelet S (2004) Aid Effectiveness and the Millennium Development Goals. Washington: Center for Global Development, Working Paper 39.

8. Lu CL, Michaud CM, Gakidou E, Khan K, Murray CJL (2006) Effect of the Global Alliance for Vaccines and Immunisation on diphtheria, tetanus, and pertussis vaccine coverage: an independent assessment. Lancet 368:1088-1095.

9. Porter ME, Teisberg EO (2006) Redefining Health Care: Creating value-based competition on results. Cambridge, MA: Harvard Business School Press.

10. Global Fund to Fight AIDS, Tuberculosis and Malaria (2006) Monitoring and evaluation tookit: HIV/AIDS, tuberculosis, and malaria: second edition. Geneva: Global Fund to Fight AIDS, Tuberculosis, and Malaria.

11. WHO (2000) The world health report 2000: health systems: improving performance. Geneva: WHO.

12. WHO (2006) The world health report 2006: working together for health. Geneva: WHO.

13. Global Fund to Fight AIDS, Tuberculosis and Malaria (2005) Making performance based funding work: mid-year results report. Geneva: Global Fund to Fight AIDS, Tuberculosis, and Malaria.

14. Feachem RG, Sabot OJ (2006) An examination of the Global Fund at 5 years. Lancet 368:537-40.

15. UNAIDS (2005) The “Three Ones” in action: where we are and where we go from here. Geneva: UNAIDS.

16. UNAIDS (2005) From advocacy to action: a progress report on UNAIDS at country level. Geneva: UNAIDS.

17. Vasan A, Hoos D, Mukherjee JS, Farmer PE, Rosenfield AG, et al. (2006) The pricing and procurement of antiretroviral drugs: an observational study of data from the Global Fund. Bull World Health Organ 84:393-8.

18. Ahmad K (2006) Global Fund suspends grants to Burma. Lancet Infect Dis 6:14.

19. Bass E (2005) Uganda is learning from its Global Fund grant suspension. Lancet 366:1839-40.

20. Eaton L (2005) Global Fund pulls grants to Myanmar and Uganda. BMJ 331:475.

21. Eaton L (2005) Global Fund toughens stance against corruption. BMJ 331:718.

22. Kapiriri L, Martin DK (2006) The Global Fund Secretariat's suspension of funding to Uganda: how could this have been avoided? Bull World Health Organ 84:576-80.

23. Parfitt T (2004) Global Fund suspends payments to Ukraine. Lancet 363:540.

24. Parry J (2005) Global Fund withdraws grants to Myanmar. Bull World Health Organ 83:724-5.

25. Drager S, Gedik G, Dal Poz MR (2006) Health workforce issues and the Global Fund to fight AIDS, Tuberculosis and Malaria: an analytical review. Human Resources for Health 4:1-12.

26. Shakow A (2006) Global Fund-World Bank HIV/AIDS programs: comparative advantage study. Global Fund/World Bank.

27. Kober K, Van Damme W (2004) Scaling up access to antiretroviral treatment in southern Africa: who will do the job? Lancet 364:103-107.

28. Bhutta ZA (2006) What does absorption capacity not measure? Lancet 368: 428-30.

**Additional Statements**

The authors declare that they are employees of the Global Fund, but do not have additional conflicts of interest.

Low-Beer and Komatsu contributed to the writing of the article, analysis and design, Afkhami, Banati, Katz, Schumacher, Sempala contributed to the analysis and design, and Cutler, Schwartlander, Tran-Ba-Huy to the design of the work.

|  | **Results for**  **1 Jun 2007** | **Percent increase from 1 Jun 2006** |
| --- | --- | --- |
| **HIV**:  People on ARV treatment | 1,100,000 | 100% |
| **TB**:  People treated under DOTS | 2,800,000 | 100% |
| **Malaria**:  Insecticide-treated nets distributed | 30,000,000 | 165% |

Figure 1: a)

Figure 1: b)

Figure 2

Figure 3

| Categories | Dollar Amount (USD) | Proportion of Original Total |
| --- | --- | --- |
| Proposal Amount at the start of the grant | 2,564,618,585 | 100.0% |
| Amount allocated after performance based funding evaluation of the first phase of implementation | 2,219,202,705 | 86.5% |
|  |  |  |
| Terminated Grants | 104,893,133 | 4.1% |
| Budget Cuts | 240,542,747 | 9.4% |
| Overall Reallocated Funds | 345,435,880 | 13.5% |

Figure 4

Figure 5

Figure 6

Figure 7
