# Supplementary material for: Making Performance-Based Funding Work for Health
Source: PLoS Med. 2007 Aug 21;4(8):e219. doi: 10.1371/journal.pmed.0040219 (PMC1949844; doi:10.1371/journal.pmed.0040219)
Supplement: Supplementary file 2 [file pmed.0040219.sd003.doc]

**FORUM SUR LES POLITIQUES DE FINANCEMENT**

**Efficacité du financement basé sur la performance dans le secteur de la santé**

**Daniel Low-Beer^*^, Houtan Afkhami, Ryuichi Komatsu, Prerna Banati, Musoke Sempala, Itamar Katz, John Cutler, Paul Schumacher, Ronald Tran-Ba-Huy, Bernhard Schwartländer**

**Financement:** Les auteurs n’ont reçu aucun financement particulier pour cet article

**Déclaration d’intérêt:** Les auteurs déclarent qu’ils sont employés du Fond Mondial

**Citation:** Low-Beer D, Afkhami H, Komatsu R, Banati P, Sempala M, et al. (2007) Making performance-based funding work for health. PLoS Med 4(8): e219. doi:10.1371/journal.pmed.0040219

**Copyright:** © 2007 Low-Beer et al. This is an open-access article distributed under the terms of the Creative Commons Attribution License, which permits unrestricted use, distribution, and reproduction in any medium, provided the original author and source are credited.

**Abréviations:** MII, moustiquaires imprégnées d'insecticide; ODM, Objectifs du Millénaire ; TB, tuberculose

**Adresse**: Performance Evaluation and Policy Unit, The Global Fund to Fight AIDS, Tuberculosis and Malaria, Vernier, Geneva, Switzerland.

* Pour toute correspondance. E-mail: daniel.low-beer@theglobalfund.org

Les financements pour l’aide au développement ont considérablement augmenté depuis 2000, notamment dans le secteur de la santé [1,2]. Au fur et à mesure que ces fonds sont utilisés par les programmes nationaux, des questions se posent quant à la façon d'accroître l'efficacité des aides financières dans le secteur de la santé.

Le financement basé sur la performance a été mis en place, notamment par le Fonds mondial de lutte contre le SIDA, la tuberculose et le paludisme (Fonds Mondial), pour inciter les pays à obtenir des résultats. Toutefois, ce type de financement est controversé du fait qu’il pourrait pénaliser les pays les plus pauvres et ne pas être assez flexible pour contribuer aux systèmes de santé de façon globale. Ces préoccupations soulèvent deux questions importantes : 1) quelle est la performance des programmes nationaux selon les différents niveaux de développement, de capacité des systèmes de santé, et de magnitude des épidémies ? 2) quels sont les défis à relever pour que le financement basé sur la performance permette une utilisation efficace des fonds, le renforcement des systèmes de santé, et l’accomplissement des Objectifs du Millénaire (ODM) ?

**Le financement basé sur la performance : Le Fonds mondial**

À titre d'exemple de financement basé sur la performance, le Fonds Mondial a décaissé 3,24 milliards de USD pour financer des programmes de lutte contre le SIDA, la tuberculose et le paludisme dans 130 pays, en utilisant ce mode de financement pour évaluer les résultats des programmes et gérer les subventions. Le financement n’est pas garanti, mais est accordé sur la base de résultats démontrés, mesurés par rapport à des objectifs et indicateurs proposés par le pays et stipulés dans l'accord de subvention initial.

Le fichier annexe S1 fournit une analyse approfondie des résultats de 370 subventions actives pour la lutte contre le SIDA, la tuberculose et le paludisme dans 130 pays, par prestation de services, par région, par type de bénéficiaire, et par caractéristiques du pays. Un deuxième fichier, S2, fournit un tableau contenant des données-clefs sur les subventions par note de performance, par caractéristiques des bénéficiaires, par maladie, et par données financières. L'analyse figurant dans le fichier S1 a permis de déterminer si le financement en fonction des résultats avait pénalisé les pays les plus pauvres ou ceux avec des systèmes de santé plus faibles. Elle reflète un accroissement rapide des prestations et traitements offerts aux personnes dans le besoin. Grace à ces financements basés sur la performance, ces services ont doublé chaque année fournissant un traitement contre le VIH à 1,100,000 personnes, un traitement contre la tuberculose à 2.8 millions de personnes et distribuant des moustiquaires imprégnées d'insecticide (MII) à 30 millions de personnes, pour protéger les familles contre le paludisme.

De surcroît, 75 % des programmes nationaux ont atteint leurs objectifs et les fonds ont été utilisés efficacement pour fournir des services de lutte contre le SIDA, la tuberculose ou le paludisme. 21 % des subventions ont obtenus des résultats inadéquats mais ont toutefois démontré un potentiel d'accélération dans leur mise en œuvre et dans la réalisation de leurs objectifs. En revanche, 4 % des subventions ont obtenu des résultats insuffisants. Les performances en Afrique subsaharienne ne sont pas inférieures à celles des autres régions. Il en est de même pour le tiers des pays les plus pauvres ; ceci démontre bien que le financement basé sur la performance fonctionne, pour autant que les objectifs soient proposés par les pays et définis par rapport à leur contexte national.

Il ressort de cette analyse que le financement basé sur la performance incite à l’accroissement rapide des activités de lutte contre les trois maladies, en reliant les fonds aux prestations de services. Pour que ne soient pas pénalisés les pays les plus pauvres, les États fragiles et ceux dont les systèmes de santé sont les plus faibles, il est essentiel que les objectifs soient fixés par les pays, et non imposés à l'échelle internationale. Cependant, la réussite du financement basé sur la performance pose de nombreux défis, notamment quant à la façon d'assurer un financement "diagonal" suffisamment souple pour que les initiatives "verticales" de lutte contre le SIDA, la tuberculose et le paludisme puissent également soutenir les systèmes de santé généraux.

**Qu’est-ce que le financement basé sur la performance ?**

Résultant de la volonté d'assurer l'utilisation efficace des fonds dans le secteur de la santé, le financement est conditionné à l’identification des problèmes, à la performance et à l’obtention de résultats par les pays. Le financement basé sur la performance s'est développé dans les années 70 dans le secteur de l'éducation, et est utilisé dans le cadre d'initiatives récentes d’aide au développement, notamment par l'alliance GAVI (Global Alliance for Vaccines and Immunisation), le Millennium Challenge Account (voir http://www.whitehouse.gov/infocus/developingnations/millennium.html), la Commission Européenne et, de façon plus générale, dans le secteur de la santé [3-6].

Tous les financements octroyés par le Fonds Mondial reposent sur le principe du financement basé sur la performance. Ces fonds sont décaissés progressivement en fonction de résultats obtenus par rapport aux objectifs, avec une explication des écarts, de ce qui a bien fonctionné et de ce qui peut être amélioré. Des notes de performance, attribuées à chaque subvention, sont utilisées comme base de décision, notamment pour : (1) arrêter des subventions (par exemple, au Nigeria, en Afrique du Sud, au Sénégal et au Pakistan) ; (2) réduire les apports de fonds (par exemple, la subvention pour le paludisme du Kenya a été réduite de 5,8 millions de USD en raison d’un retard d'une année dans la mise en œuvre ) ; ou (3) accélérer le financement, comme en témoigne la subvention pour le paludisme de l'Éthiopie, qui a vu son budget de la quatrième année être réaffecté à la troisième année pour doubler le nombre de MII (moustiquaires imprégnées d’insecticides) distribuées aux familles (Encadré 1). Les décaissements sont effectués suivant la rapidité et l'efficacité de la mise en oeuvre, et non selon un échéancier déterminé. Si des programmes progressent plus lentement que prévu, les fonds inutilisés peuvent être réaffectés à d’autres subventions, et les besoins d'assistance technique signalés. Si la mise en oeuvre est plus rapide que prévu, le décaissement des fonds peut être accéléré.

Le financement basé sur la performance repose fondamentalement sur la responsabilité des pays en ce qui concerne les objectifs et la mise en  œuvre ; l'interférence du Fonds Mondial étant limitée. Les plans de mise en oeuvre et les objectifs sont proposés par les pays (sans implication formelle du Fonds Mondial), acceptés par des représentants du gouvernement, de la société civile et des personnes affectées par les maladies dans le pays (en tant que parties d'une instance de coordination nationale) et examinés par un groupe international d’experts techniques. La performance se mesure par rapport à ce qui peut être réellement mis en œuvre par un pays sur une période donnée. La Russie, par exemple, n’est pas comparée directement à la Zambie, ni l’Inde au Swaziland. La responsabilisation des pays et le financement basé sur la performance visent à renforcer la gestion des programmes au niveau national. Comme l'a souligné le responsable des programmes du Guatemala : « Si les maîtres d’œuvre voient leurs résultats chuter et passer dans le rouge, ils nous téléphonent et nous en discutons pour trouver des solutions ; tout aussi important, nous rendons compte de notre performance et nous avons une discussion approfondie, pas seulement sur les aspects politiques mais sur des questions concrètes, telles que la mise en oeuvre et l’avancement. » [5].

**Les pays peuvent-ils utiliser efficacement les subventions ?**

Les performances des programmes financés pour la lutte contre le SIDA, la tuberculose et le paludisme sont intrinsèquement variables et empreintes de risques. On s’attend souvent à ce que le financement "achète" les résultats (par exemple, pour la réalisation des ODM, chiffrés à 189 milliards de USD d’ici 2015 [7]) ; cependant, les risques et les retours n'apparaissent souvent que lors de la mise en oeuvre des programmes. Comme le montre l'analyse du Fonds Mondial (fichier S1), les programmes affichant d'excellents résultats (notés A) ont atteint 120 % de leurs objectifs initiaux pour les principales interventions, contre 60 % pour les programmes affichant des résultats inadéquats (notés B2). Baser l'assistance financière et technique sur les écarts de performance peut accélérer la mise en oeuvre et doubler les résultats des subventions de programmes de santé.

75 % des programmes ont généré des résultats satisfaisants ou excellents, 21 %, des résultats inadéquats, et 4 %, des résultats insuffisants (Figure 1). Les décisions les plus difficiles à prendre concernaient les 21 % de subventions notées B2, car il fallait peser le pour et le contre, entre des résultats médiocres par rapport aux objectifs fixés et la preuve d'un potentiel d'accélération dans la mise en oeuvre. Ensemble, les 215 programmes évalués sur les 18 premiers mois de leur mise en oeuvre ont atteint 94 % de leurs objectifs (Figure 2). Certains d'entre eux n'ont pas atteint leurs objectifs, mais ceux qui se sont avérés performants ont permis au Fonds Mondial d'atteindre ses objectifs globaux. Ces programmes performants permettent au Fonds Mondial d'absorber un certain niveau de risque (par exemple, des performances médiocres pour les premiers investissements au Nigeria, en Ouganda et au Kenya), tout en enregistrant globalement des résultats positifs. Des points faibles sont apparus tels que des retards dans le démarrage d'interventions pour la lutte contre le paludisme, dus au fait que quelques subventions importantes n'ont pas été utilisées suffisamment rapidement pour distribuer des MII afin de protéger les familles, notamment en Éthiopie, au Kenya et en Tanzanie. Toutefois, l'exemple de l'encadré 1 montre qu'une subvention dont le démarrage est lent peut rattraper rapidement son retard une fois que les problèmes initiaux sont résolus et que les investissements nécessaires sont effectués. Il ressort également de cette analyse que dans de nombreuses subventions, la prévention de la transmission mère-enfant du VIH n'a pas généré les résultats escomptés. Les questions d’inégalités homme-femme doivent être abordées au plus vite pour que les femmes bénéficient de ce qui est essentiellement un "vaccin" abordable afin d’empêcher que les enfants soient contaminés. Malgré de nombreux défis à relever, la plupart des pays utilise efficacement les fonds qui leur sont alloués.

**Enseignements tirés des performances : une mise en œuvre intelligente**

Le financement basé sur la performance fait apparaître nombre de problèmes de mise en oeuvre. Il permet ainsi aux pays et partenaires d'y remédier de façon transparente, tout en favorisant l'indispensable "apprentissage par la pratique". Plusieurs enseignements découlent des résultats des programmes de lutte contre les trois maladies.

Comme le montre l'analyse du fichier S1, la société civile a été un maître d'oeuvre particulièrement efficace (83 % affichant de solides performances), ceci en plus de son rôle reconnu de gouvernance et de supervision. Les programmes de lutte contre la tuberculose ont également généré de bons résultats (84 % se sont avérés très performants). Ces résultats sont liés au soutien important du partenariat Halte à la Tuberculose dans la coordination des partenaires, ainsi que dans la mise à disposition d'une assistance technique et d'une aide à la gestion des programmes. Ce rôle de soutien et de coordination des partenaires peut être un enseignement pour le VIH et le paludisme. L'assistance technique a été surtout nécessaire lorsque les pays ont accéléré la mise en œuvre des programmes, par exemple lorsque le Malawi et l'Éthiopie ont étendu des services performants à des zones rurales. Cette assistance n'est pas seulement nécessaire lorsque des problèmes surgissent. Les retombées potentielles les plus importantes de l'assistance technique résultent d'un ensemble général de services visant à soutenir les programmes performants (ainsi que les programmes posant des problèmes). S'ils sont efficaces, ils peuvent améliorer les résultats de 90 % en Afrique sub-saharienne. Comme mentionné, les performances du tiers le plus pauvre des pays ne sont pas moindres que celles des pays les plus riches. 9 % seulement des fonds ont été réaffectés au détriment des pays les plus pauvres en raison de performances inadéquates, soit un tiers de moins que les fonds retirés de toutes les subventions. Le financement basé sur la performance peut être un investissement sur la performance et la réduction de la pauvreté.

**Conclusion : le "financement diagonal" pour soutenir les systèmes de santé**

Malgré de nombreux défis à relever, le financement basé sur la performance encourage activement la montée en puissance de la lutte contre le VIH, la tuberculose et le paludisme. Il a également des répercussions plus générales sur l’apport de fonds pour le développement et la santé. Utilisé de manière optimale, le financement basé sur la performance permet d’inscrire l’inventivité de solutions nationales dans un cadre très précis axé sur la performance, assurant une mise à disposition rapide de services aux personnes qui en ont besoin. La plupart des programmes nationaux peuvent utiliser efficacement les fonds qui leur sont alloués, dans les pays pauvres et les États fragiles, pour lutter contre les trois maladies et avec des maîtres d'oeuvre de la société civile et gouvernementaux. Il existe toutefois un risque inhérent face aux résultats que nous pouvons espérer des programmes de santé. La nature variable des retombées des programmes de santé (les programmes performants génèrent deux fois plus de résultats) n'est pas toujours reconnue et doit être gérée activement, notamment avec des incitations financières et une assistance technique.

Une question majeure est de savoir si le financement pour des maladies spécifiques telles que le SIDA, la tuberculose et le paludisme (financement vertical) a des effets positifs ou négatifs sur les systèmes de santé plus généraux et horizontaux. Le Fond Mondial fournit un « financement diagonal » avec des objectifs très précis en vue d’atteindre les résultats contre les maladies tout en permettant de financer plus largement le renforcement du système de santé. À mesure que les programmes de santé montent en puissance, les principaux obstacles au renforcement des systèmes de santé deviennent de plus en plus évidents. Un tel renforcement nécessite des fonds spéciaux et davantage de souplesse dans le financement des programmes spécifiques de lutte contre les maladies pour pallier aux principales carences en matière de capacités. Grace à la flexibilité du financement du Fonds Mondial pour le soutien des services et des systèmes, les pays ayant des systèmes de santé ou des capacités en ressources humaines plus faibles n'ont pas obtenu des résultats moindres que les autres (25 % et 26 % de pays aux performances médiocres, contre 25 % pour toutes les subventions). Les subventions du Fonds mondial peuvent être octroyées "verticalement" aux programmes de lutte contre le SIDA, la tuberculose et le paludisme, ou "horizontalement", via un fonds commun ou SWAp (par exemple, au Ghana ou en Mozambique) De nombreux programmes efficaces ont utilisé les subventions du Fonds Mondial de manière "diagonale" pour financer des services de lutte contre le SIDA, la tuberculose et le paludisme, tout en mettant en place les infrastructures nécessaires, y compris les ressources humaines requises.

Sur le terrain, la flexibilité du financement est essentielle. En Éthiopie, lors de l'extension de services de lutte contre le SIDA et le paludisme à des zones rurales, il a fallu former 30 000 professionnels et réhabiliter des postes de santé communautaires, le tout financé par le Fonds Mondial. À Haïti, une étude de 14 mois a montré qu’un programme de lutte contre le SIDA financé par le Fonds Mondial a engendré une hausse des vaccinations, des consultations prénatales, du planning familial et de l’approvisionnement de médicaments essentiels, ce qui a amélioré le moral du personnel et la capacité des centres de santé [8]. Toutefois, de nombreux programmes n'utilisent pas les subventions du Fonds Mondial de manière aussi souple et efficace, et nous devons en tenir compte dans le financement des systèmes de santé. La réalisation des ODM nécessite d'investir 67 milliards de USD dans le personnel de santé [7]. D'autres bailleurs de fonds sont urgemment requis, surtout pour le financement d'une infrastructure de santé à long terme [9]. La réalisation des ODM exige une augmentation considérable des fonds, ainsi que des mécanismes innovants pour assurer leur utilisation efficace. Le financement basé sur la performance peut constituer, à lui seul, un mécanisme important pour fournir un cadre précis et des incitations à la mise en œuvre, ainsi que pour gérer les risques liés au financement des programmes de lutte contre le SIDA, la tuberculose et le paludisme, ainsi que des systèmes de santé.

Informations annexes

Texte S1. Performance des résultats communiqués au Fonds mondial par rapport aux objectifs, pour les 370 subventions actives du Fonds mondial à fin 2006.

Texte S2. Tableau des données essentielles concernant les subventions examinées, incluant leurs notes, les caractéristiques des récipiendaires principaux, la maladie ciblée et des informations financières.

**Contribution des auteurs:** DLB, HA, RK, PB, IK, JC et BS ont conçu l’étude. DLB, HA, RK, PB, MS, IK, et PS ont analysé les données. DLB a écrit l’article avec les contributions de RK, PB, MS, HA, PS, RTBH, et BS

**Références**

1. OMS (2005) - Santé et Objectifs de Développement du Millénaire. Genève : OMS.
2. Organisation de Coopération et de Développement Économiques (2006) - mise à jour concernant l'aide apportée dans le secteur de la santé, 2006. Paris : OCDE.
3. Lu CL, Michaud CM, Gakidou E, Khan K, Murray CJL (2006) - Impact de la GAVI (Global Alliance for Vaccines and Immunisation) sur la couverture des vaccins contre la diphthérie, le tétanos et la coqueluche : évaluation indépendante. Lancet 368 :1088-1095.
4. Porter ME, Teisberg EO (2006) "Redefining health care: Creating value-based competition on results" (Redéfinition des soins de santé : création d'une compétition basée sur la valeur pour l'obtention de résultats). Cambridge, MA : Harvard Business School Press.
5. Fonds mondial de lutte contre le SIDA, la tuberculose et le paludisme (2006) - Partenaires dans l'impact : rapport de résultats. Genève : Fonds mondial de lutte contre le SIDA, la tuberculose et le paludisme.
6. Fonds mondial de lutte contre le SIDA, la tuberculose et le paludisme (2002) - Le cadre de travail du Fonds mondial de lutte contre le SIDA, la tuberculose et le paludisme : nom, objectif, principes et champ d'application du fonds. Genève : Fonds mondial de lutte contre le SIDA, la tuberculose et le paludisme.
7. UN Millennium Project (2005) – "Investing in development: A practical plan to achieve the Millennium Development Goals" (Investir dans le développement : plan pratique pour atteindre les Objectifs de Développement du Millénaire). Overview (Présentation générale). New York.
8. Walton DA, Farmer PE, Lambert W, Leandre F, Koenig SP, et al (2004) – "Integrated HIV prevention and care strengthens primary health care: lessons from rural Haiti" (La prévention et les soins intégrés pour la lutte contre le SISA renforcent les soins de santé de base : les enseignements tirés des zones rurales de Haïti). J Public Health Policy 25:137-58.
9. Shakow A (2006) – "Global Fund-World Bank HIV/AIDS programs: comparative advantage study" (Programmes de lutte contre le VIH/SIDA du Fonds mondial et de la Banque mondiale : étude comparative des avantages). Global Fund/World Bank.

**Encadré 1 : Exemple de financement basé sur la performance dans un pays : subvention pour la lutte contre le paludisme en Éthiopie**

En juin 2005, le ministère de la santé de l'Éthiopie n'avait pas encore fourni la moindre MII financée par la subvention, malgré un objectif de deux millions de MII censées avoir été distribuées à cette date. Cette situation était principalement due à des goulets d'étranglement dans l'approvisionnement et des problèmes du côté de l'offre mondiale, à la fois pour les MII longue durée et les combinaisons thérapeutiques antipaludiques à base d'artémisinine (CTA). Le Fonds mondial a alors imposé une condition très stricte pour la poursuite du financement : distribuer les MII avant la saison suivante de paludisme.

Ce délai conditionnel a incité le pays à trouver des solutions innovantes. L’Éthiopie a demandé une assistance technique à l’UNICEF, renforcé les capacités de ses services d'achats, et investi pour éliminer les retards dans la chaîne logistique nationale. Le résultat : deux millions de MII longue durée furent distribuées en 4 mois, avant le début de la saison de paludisme. Le ministère de la santé réussit également à former 4 416 auxiliaires de santé à l'utilisation des MII et 5 222 au diagnostic et au traitement du paludisme, et distribua 2 millions de doses de CTA.

Compte tenu de cette montée en puissance, il est maintenant prévu de distribuer 7 millions de MII supplémentaires, conformément aux objectifs d'Abuja concernant la lutte contre le paludisme (voir http://www.rbm.who.int/docs/abuja_declaration.pdf) et aux ODM. Face au niveau de performance actuel de l'Éthiopie, son budget pour la quatrième année de subvention a été réaffecté sur la troisième année. C'est en fait un bon exemple des subventions relatives au paludisme dont le démarrage peut être lent, mais dont les résultats peuvent s’accélérer rapidement lorsque les problèmes initiaux ont été résolus. Le ministère de la santé éthiopien a livré les commentaires suivants à propos des incitations du financement basé sur la performance : *« La différence est due au fait que vous nous avez clairement avertis que nous étions en zone rouge et que nous pouvions perdre notre argent si nous n'obtenions pas de résultats. Nous avons examiné la question, nous pouvions pousser l’analyse et nous avons tous deux vu le problème... et c’est l’ajustement que nous avons opéré qui a permis d’obtenir les résultats. Le financement en fonction des résultats nous a aidé a redéfinir la mise en oeuvre »* [5].

Figure 1 - Écarts de performance de 215 subventions évaluées au 1^er^ décembre 2006.

A : objectifs atteints ou dépassés, B1 : résultats satisfaisants, B2 : résultats insatisfaisants mais preuve de potentiel, C : résultats inacceptables

Figure 2. Écarts de performance programmatique relatifs aux principaux domaines de prestation de services pour le traitement du VIH, la prévention et les soins, le traitement de la tuberculose sous DOTS et de la tuberculose polypharmacorésistante (MDR-TB), la prévention (MII) et le traitement du paludisme, ainsi que l'appui médico-social transversal et la formation des ressources humaines.
